# The association between depressive symptoms and masked hypertension in participants with normotension measured at research center

**DOI:** 10.1038/s41440-023-01484-8

**Published:** 2023-10-31

**Authors:** Sayuri Tokioka, Naoki Nakaya, Kumi Nakaya, Mana Kogure, Rieko Hatanaka, Ippei Chiba, Ikumi Kanno, Kotaro Nochioka, Hirohito Metoki, Takahisa Murakami, Michihiro Satoh, Tomohiro Nakamura, Mami Ishikuro, Taku Obara, Yohei Hamanaka, Masatsugu Orui, Tomoko Kobayashi, Akira Uruno, Eiichi N. Kodama, Satoshi Nagaie, Soichi Ogishima, Yoko Izumi, Nobuo Fuse, Shinichi Kuriyama, Atsushi Hozawa

**Affiliations:** 1 Tohoku University Graduate School of Medicine, Sendai, Japan; 2 Tohoku Medical Megabank Organization, Tohoku University, Sendai, Japan; 3 Tohoku University Hospital, Tohoku University, Sendai, Japan; 4 Tohoku Medical and Pharmaceutical University, Sendai, Japan; 5 Kyoto Women’s University, Kyoto, Japan; 6 International Research Institute of Disaster Science, Tohoku University, Sendai, Japan

**Keywords:** depression, home blood pressure, hypertension, masked hypertension, office blood pressure

## Abstract

Masked hypertension is a risk factor for cardiovascular diseases. However, masked hypertension is sometimes overlooked owing to the requirement for home blood pressure measurements for diagnosing. Mental status influences blood pressure. To reduce undiagnosed masked hypertension, this study assessed the association between depressive symptoms and masked hypertension. This cross-sectional study used data from the Tohoku Medical Megabank Project Community-Based Cohort Study (conducted in Miyagi Prefecture, Japan, from 2013) and included participants with normotension measured at the research center (systolic blood pressure<140 mmHg and diastolic blood pressure <90 mmHg). Depressive symptoms were assessed using the Center for Epidemiologic Studies Depression Scale (Japanese version). Masked hypertension was defined as normotension measured at the research center and home hypertension (home systolic blood pressure ≥135 mmHg or home diastolic blood pressure ≥85 mmHg). The study comprised 6705 participants (mean age: 55.7 ± 13.7 years). Of these participants, 1106 (22.1%) without depressive symptoms and 393 (23.2%) with depressive symptoms were categorized to have masked hypertension. Sex-specific and age-adjusted least mean squares for home blood pressure, not for research blood pressure were significantly higher in the group with depressive symptoms in both sex categories. The multivariate odds ratio for masked hypertension in the patients with depressive symptoms was 1.72 (95% confidence interval: 1.26–2.34) in male participants and 1.30 (95% confidence interval: 1.06–1.59) in female ones. Depressive symptoms were associated with masked hypertension in individuals with normotension measured at the research center. Depressive symptoms may be one of the risk factors for masked hypertension.

**Figure:**
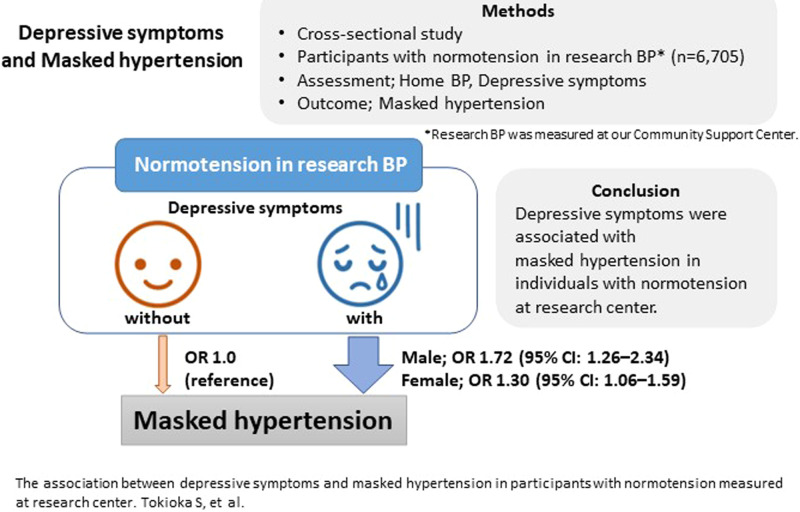
Depressive symptoms were associated with masked hypertension in individuals with normotension measured at research center.

Depressive symptoms were associated with masked hypertension in individuals with normotension measured at research center.

## Abstract (web summary)

Depressive symptoms were associated with masked hypertension in individuals with normotension measured at research center.

## Introduction

Blood pressure (BP) levels can be categorized into four patterns according to office BP and home BP—namely: normotension (office normotension and home normotension), white-coat hypertension (WCHT; office hypertension [HT] and home normotension), masked hypertension (MHT; office normotension and home HT), and sustained HT (office HT and home HT) [1–4]. Sustained HT, WCHT, and MHT are all risk factors for cardiovascular diseases (CVDs) [1–4]. Individuals with MHT were reported to have a higher prevalence of organ damage and a twofold higher risk of cardiovascular events than did normotensive ones [5, 6]. MHT management is essential for CVD prevention. Nonetheless, MHT is likely to be overlooked because office BP is within the normal limit and home BP measurement is required for diagnosing MHT [7].

Previous studies showed that high normal office BP, male sex, smoking, diabetes, and antihypertensive treatment were risk factors for MHT [1–4, 8]. Individuals with risk factors for MHT are recommended to measure their home BP for MHT screening, even if office BP measurement suggests that they are normotensive [1–4].

Mental status, including depression, anxiety, stress, and personality, reportedly could influence BP [9–13]. However, the association between MHT and depression remains unclear. Two previous studies assessed the association between depression and MHT; nevertheless, the evidence is still considered inconclusive because the analysis was performed without considering enough covariates, and included only those with treated HT [14, 15]. Although the risk of MHT is reported to be higher in patients receiving antihypertensive therapy [1–4, 8], MHT in those without treated HT also requires treatment, indicating the need for further studies that could include patients with and without treated HT.

We hypothesized that depressive symptoms could be related to MHT for two reasons. First, individuals with depressive symptoms tend to adopt an unhealthy lifestyle, including diet, drinking, smoking, and poor medical adherence [16]. Second, mental status, which influences BP, varies throughout the day [17], and may result in differences between office and home BP.

The present study aimed to assess the association between depressive symptoms and MHT to classify the risk for MHT in individuals with normotension using BP measured at the research center. If the results support our hypothesis, depressive symptoms may be one of the risk factors for MHT.

Point of view

- Clinical relevance:
  Depressive symptoms were associated with masked hypertension in individuals with normotension using research blood pressure, and it suggested that depressive symptoms may be one of the risk factors for masked hypertension.
- Future direction:
  A prospective study to evaluate the relationship between depressive symptoms and masked hypertension is warranted.
- Consideration for the Asian population:
  Both of depression and hypertension is increasing in Asia. Assessment of depressive symptoms in clinical settings might reveal masked hypertension, and it might contribute to reduce incidence of cardiovascular disease or mortality in Asia.

## Methods

### Study participants

This cross-sectional study used data from the Tohoku Medical Megabank Project Community-Based Cohort Study (TMM CommCohort Study), a community-based prospective cohort study conducted in Miyagi Prefecture, Japan [18]. In the TMM CommCohort Study, the baseline survey was conducted from 2013 to 2016. Participants were recruited through three major approaches. First, the specific health checkup sites-based survey (Type 1 survey) collected basic information, including blood and urine, a questionnaire, and municipal health check-up data at the sites of the annual community health examination. Second, Type I additional survey was conducted in places selected by the municipality and the TMM on dates that differed from the specific health checkups in the municipality. Third, the Community Support Center-based survey (Type 2 survey) was conducted at the Community Support Center with physical examination, blood and urine tests, and detailed measurements. Some individuals who participated in the Type 1 survey or Type 1 additional survey, visited the Community Support Center and underwent detailed measurements similar to those in the Type 2 survey. This study was conducted in accordance with the principles embodied in the Declaration of Helsinki and was approved by the Institutional Review Board of the Tohoku Medical Megabank Organization (approval number: 2022-4-160). All participants provided written informed consent before their participation in this study.

The eligible criteria were: (i) participation in Type 1 or Type 1 additional with detailed measurement, or Type 2 surveys of the TMM CommCohort Study; (ii) not having HT, which was defined as systolic BP (SBP) ≥ 140 mmHg and/or diastolic BP (DBP)≥ 90 mmHg using BP measured at the research center; (iii) having home BP measurements for at least three days for two weeks [19, 20]; and (iv) undergoing complete assessment using the Japanese version of the Center for Epidemiologic Studies Depression Scale (CES-D). Those who withdrew from the study by October 5, 2021 (*n* = 231), those who did not return self-report questionnaires (*n* = 16), those with missing data of BP and CES-D (*n* = 5,369), and those who underwent BP measurements at the Community Support Center more than 1 month after CES-D assessment (*n* = 2,666) were excluded to assess the association between depressive symptoms and BP within a short period.

### Data collection and measurement at the baseline survey

#### CES-D and definition of depressive symptoms

The CES-D self-reporting questionnaire was used for assessing depression [21–23]. The CES-D comprised 20 items (16 positive statements and 4 negative statements), with each item ranked from 0 to 3. A cutoff score of 15/16 is widely applied to screen for depression in Japan [24]. In the present study, depressive symptoms were defined as CES-D scores ≥16.

#### Research BP

We defined BP and HR measured at our Community Support Center as research BP and research HR, respectively [25]. After the participants had rested for 1–2 min, a trained nurse measured research BP twice in the seated participants using an electronic upper arm cuff device (HEM-9000AI; OMRON Corp., Kyoto, Japan) [25]. The mean of the two BP values was analyzed.

#### Home BP

The participants measured their home BP and HR every morning and evening for two weeks. In accordance with the Japanese guidelines, morning BP was measured in the sitting position within 1 hours of waking and after 1–2 minutes of rest before the participants took their medicines and ate breakfast [1]. Evening BP was measured in the sitting position after 1–2 minutes in the evening. An electronic upper arm cuff device (HEM-7080IC; OMRON Corp.) was provided for home BP measurement. The morning and evening BP values for two weeks were used for calculating each average that was included in the analysis.

### Physical examinations, laboratory data, and questionnaires

Physical examinations included measurements of height (AD-6400; A&D Co, Ltd, Tokyo, Japan), weight (InBody720; Biospace Co, Ltd, Seoul, Republic of Korea), body mass index (BMI), research SBP and DBP, and heart rate (HR). The following blood test data were extracted: γ-glutamyl transpeptidase, hemoglobin A1c (HbA1c), total cholesterol, low-density lipoprotein cholesterol, high-density lipoprotein cholesterol, triglycerides, and creatinine. The daily NaCl intake was estimated using Tanaka’s method [26].

Lifestyle habits including smoking, drinking, and exercise, medical history, use of sleeping pills, and educational background were defined using self-report questionnaires. Smoking status was categorized into people who never smoked, those who formally smoked, and individuals who smoke daily. Alcohol intake per day was calculated based on drinking habits (including the type of drink, frequency, and amount) as follows: the frequency in a week of alcohol consumption was multiplied by the amount of alcohol consumed in a single occasion, and the product was subsequently divided by 7. Regarding exercise, the participants were asked how often and how long they performed several exercises according to their intensity. Regular exercise was defined as the performance of any type of exercise at least once per week. Among the several items of self-reported medical history, data on HT, diabetes mellitus, dyslipidemia, stroke, heart failure, myocardial infarction, and depression were collected. When they answered that they regularly visited the clinic for HT, diabetes mellitus (DM), or dyslipidemia, they were regarded to have treated HT, treated DM, or treated dyslipidemia. Users of sleeping pills were defined as those who used sleeping pills at least once a week. Education status was categorized into junior high school, high school, vocational college, junior college, university, graduate school, and other.

Additionally, the examination year and extent of house damage caused by the Great East Japan Earthquake in 2011 were considered because the Great East Japan Earthquake might have physically and mentally affected the participants. The extent of house damage was categorized into six degrees, ranging from no damage to totally damaged. The season at the time of examination was also included because it could influence BP [27] and mood disorders [28]. The season of the examination date was defined according to the mean temperature in Miyagi prefecture: winter (December, January and February); summer, from July to September; and spring or fall, others.

### MHT

The study outcome was MHT, which was based on BP categories of research BP and home BP, regardless of the presence or absence of treated HT. MHT was defined as not meeting the criteria for HT (SBP ≥ 140 mmHg or DBP ≥ 90 mmHg) using research BP but meeting the criteria for home HT (SBP ≥ 135 mmHg or DBP ≥ 85 mmHg) on home BP measurement, according to the guidelines [1–4]. When either the morning or evening BP value met the criteria for MHT, it was considered MHT.

### Statistical analysis

Data are presented as means±standard deviations for continuous variables with normal distribution or medians (25th–75th percentile) for continuous variables with skewed distribution and as numbers (percentages) for categorical variables. The sex-specific characteristics of the participants with and without depressive symptoms were compared using the *t*-test for normal distribution (Student’s *t*-test for two groups with similar variance, and Welch’s *t*-test for two groups with dissimilar variance), the Mann–Whitney U test for skewed distribution, and the chi-squared test for categorical variables. Sex-specific and age-adjusted least mean squares were calculated for BP and HR. Regarding the evaluation of the association between depressive symptoms and MHT, multivariate logistic regression models were used to obtain the odds ratios (ORs) and 95% confidence intervals (CIs). A complete case analysis using three models was performed—namely, (i) model 1, which included age and sex in all-analysis and which included age in sex-specific analysis; (ii) model 2, which included model 1, BMI, research SBP, research HR, treated HT, HbA1c, low-density lipoprotein cholesterol, smoking status, alcohol intake, NaCl intake, and regular exercise; and (iii) model 3, which included model 2, use of sleeping pills, extent of house damage, educational status, season of the examination date, and examination year.

A subgroup analysis specified by sex was performed according to age, research SBP, treated HT, and medical history of depression. Age category was divided into four according to the quartiles. Cutoff values of research SBP were median rounded off. The interaction of each category with depressive symptoms was analyzed by adding variables and multiplying depressive symptoms and categories to the model. Age and research SBP were analyzed as continuous variables.

Two-tailed *P*-values < 0.05 were considered statistically significant. All analyses were performed using R version 4.2.1 for Linux.

## Results

**Fig. 1:**
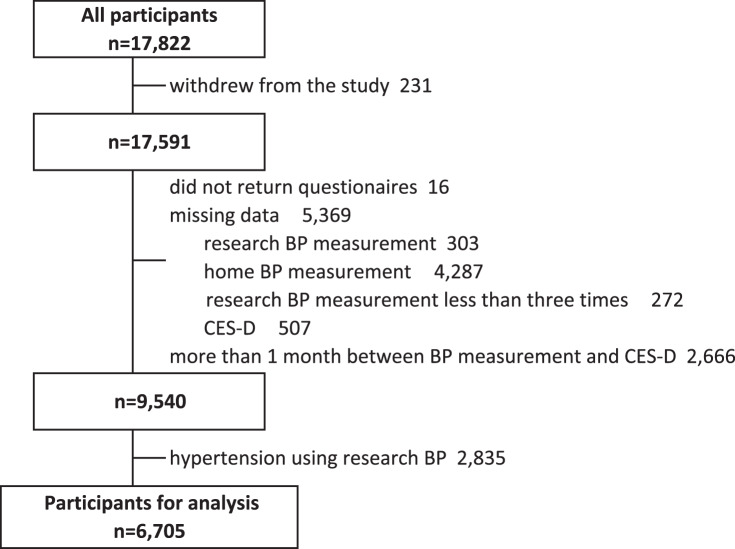
Flow of participant selection. BP blood pressure, CES-D Center for Epidemiologic Studies Depression Scale

A total of 6705 participants (female, 74.9%; mean age, 55.7 years) without HT using research BP were included in the analysis (Fig. 1). Table 1 summarizes the characteristics of the participants according to depressive symptoms in each sex. Overall, 310 (18.4%) of the male and 1384 (27.7%) of female participants had depressive symptoms. The group with depressive symptoms was younger (55.3 years versus 60.1 years in male participants, 52.3 years versus 55.4 years in female ones), and had a lower research SBP (123.7 mmHg versus 124.3 mmHg in male individuals, 117.5 mmHg versus 118.9 mmHg in female participants) than did the group without depressive symptoms. Univariate analysis showed no significant differences in home BP and medical history of atherosclerotic diseases, including HT, between the two groups.

**Table 1:** Sex-specific participant characteristics according to depressive symptoms

	Male			Female		
	Non-depressive symptoms	Depressive symptoms	*P*-value	Non-depressive symptoms	Depressive symptoms	*P*-value
	(*n* = 1,375)	(*n* = 310)		(*n* = 3,636)	(*n* = 1,384)	
Age (years), mean (SD)	60.1 (13.7)	55.3 (15.7)	<0.001	55.4 (12.9)	52.3 (14.1)	<0.001
BMI, mean (SD)	23.4 (2.8)	23.7 (3.4)	0.22	21.9 (3.2)	22.1 (3.6)	0.12
Research SBP (mmHg), mean (SD)	124.3 (9.6)	123.7 (9.6)	0.32	118.9 (11.8)	117.5 (11.6)	<0.001
Research DBP (mmHg), mean (SD)	75.7 (7.7)	75.9 (7.9)	0.66	73.1 (7.9)	72.9 (8.1)	0.42
Research HR (/min), mean (SD)	64.2 (9.9)	65.5 (9.9)	0.034	66.7 (8.9)	67.2 (9.4)	0.12
HbA1c (%), mean (SD)	5.6 (0.56)	5.5 (0.56)	0.43	5.5 (0.42)	5.4 (0.50)	0.098
T-chol (mg/dL), mean (SD)	198.5 (33.6)	199.7 (35.9)	0.58	212.0 (35.0)	209.0 (34.9)	0.006
LDL-chol (mg/dL), mean (SD)	118.4 (29.3)	119.8 (31.0)	0.45	125.7 (31.2)	123.3 (31.1)	0.013
HDL-chol (mg/dL), mean (SD)	57.3 (15.0)	55.8 (14.9)	0.11	68.6 (16.1)	67.5 (16.0)	0.037
TG (mg/dL), mean (SD)	97.0 (69.0–139.5)	96.0 (69.3–143.0)	0.84	75.0 (55.0–106.0)	76.0 (55.0–109.0)	0.48
γ-GTP (IU/L), mean (SD)	38.5 (38.4)	45.2 (56.7)	0.011	22.1 (17.3)	22.4 (27.1)	0.65
Cr (mg/dL), mean (SD)	0.83 (0.16)	0.83 (0.14)	0.42	0.60 (0.10)	0.61 (0.16)	0.011
NaCl intake (g/day), mean (SD)	13.3 (2.9)	13.0 (3.2)	0.10	12.2 (2.7)	12.0 (2.8)	0.028
Alcohol intake (g/day), median (IQR)	10.2 (0.7–29.5)	7.9 (0.00–32.2)	0.36	0.0 (0.0–4.6)	0.0 (0.0–4.2)	0.10
Smoking status			0.004			<0.001
Never smoked, *n* (%)	417 (30.4)	106 (34.2)		2,910 (80.2)	1,049 (76.1)	
Formally smoked, *n* (%)	732 (53.3)	135 (43.5)		503 (13.9)	206 (14.9)	
Smoke daily, *n* (%)	224 (16.3)	69 (22.3)		214 (5.9)	123 (8.9)	
Regular exercise, *n* (%)	946 (69.1)	187 (60.5)	0.005	2,467 (67.9)	785 (56.8)	<0.001
Use of sleeping pills, *n* (%)	76 (5.6)	42 (13.9)	<0.001	193 (5.4)	210 (15.5)	<0.001
*Medical history*, *n* (%)						
Treated hypertension	359 (26.1)	75 (24.2)	0.53	459 (12.6)	163 (11.8)	0.44
Treated diabetes mellitus	94 (6.8)	20 (6.5)	0.90	75 (2.1)	40 (2.9)	0.10
Treated dyslipidemia	202 (14.7)	43 (13.9)	0.78	473 (13.0)	178 (12.9)	0.93
Stroke	38 (2.8)	10 (3.2)	0.80	22 (0.6)	11 (0.8)	0.58
Myocardial infarction	72 (5.2)	16 (5.2)	1.00	50 (1.4)	20 (1.4)	0.96
Depression	26 (1.9)	19 (6.1)	<0.001	89 (2.4)	130 (9.4)	<0.001
*Home BP and HR measurement*, mean (SD)						
Morning SBP (mmHg)	127.7 (12.5)	128.6 (13.2)	0.22	120.0 (13.9)	119.9 (14.2)	0.85
Morning DBP (mmHg)	75.6 (7.9)	76.8 (9.0)	0.024	71.0 (8.4)	71.4 (8.5)	0.22
Morning HR (/min)	66.0 (8.8)	68.4 (9.7)	<0.001	67.3 (7.5)	68.7 (8.2)	<0.001
Evening SBP (mmHg)	123.9 (12.2)	125.5 (13.3)	0.045	116.5 (13.3)	116.8 (13.8)	0.58
Evening DBP (mmHg)	71.0 (8.3)	72.5 (9.3)	0.007	67.9 (8.3)	68.3 (8.6)	0.12
Evening HR (/min)	70.1 (9.1)	71.4 (9.1)	0.030	68.9 (7.4)	70.0 (7.8)	<0.001
House damage, *n* (%)			0.17			<0.001
Totally damaged	96 (7.2)	28 (9.4)		254 (7.2)	161 (12.1)	
Majorly damaged	88 (6.6)	24 (8.0)		249 (7.0)	115 (8.6)	
Half-damaged	124 (9.3)	38 (12.7)		350 (9.9)	140 (10.5)	
Partially damaged	647 (48.3)	126 (42.1)		1,542 (43.6)	556 (41.7)	
No damage	331 (24.7)	69 (23.1)		1,055 (29.8)	320 (24.0)	
Non-impaired area	54 (4.0)	14 (4.7)		88 (2.5)	42 (3.1)	
Education, *n* (%)			0.74			0.44
Junior high school	111 (8.1)	28 (9.1)		148 (4.1)	75 (5.5)	
High school	631 (46.0)	131 (42.2)		1,749 (48.3)	652 (47.4)	
Vocational school	125 (9.1)	29 (9.4)		721 (19.9)	281 (20.4)	
Junior collage	30 (2.2)	8 (2.6)		504 (13.9)	187 (13.6)	
University	420 (30.6)	95 (30.7)		456 (12.6)	167 (12.1)	
Graduate school	46 (3.4)	14 (4.5)		32 (0.9)	10 (0.7)	
Other	9 (0.7)	4 (1.3)		14 (0.4)	3 (0.2)	
Examination season, *n* (%)			0.23			0.79
Winter	420 (30.5)	84 (27.1)		1,169 (32.2)	432 (31.2)	
Spring or Fall	726 (52.8)	163 (52.6)		576 (15.8)	218 (15.8)	
Summer	229 (16.7)	63 (20.3)		1,891 (52.0)	734 (53.0)	
Examination year, *n* (%)			0.43			0.53
2013	65 (4.7)	15 (4.8)		210 (5.8)	86 (6.2)	
2014	808 (58.8)	181 (58.4)		2,096 (57.6)	789 (57.0)	
2015	445 (32.4)	107 (34.5)		1,133 (31.2)	446 (32.2)	
2016	57 (4.1)	7 (2.3)		197 (5.4)	63 (4.6)	

*BMI* body mass index, *BP* blood pressure, *Cr* creatinine, *DBP* diastolic blood pressure, *G-GTP* γ-glutamyl transpeptidase, *HDL-chol* high-density lipoprotein cholesterol, *HbA1c* hemoglobin A1c, *HR* heart rate, *IQR* interquartile range, *LDL-chol* low-density lipoprotein cholesterol, *MHT* masked hypertension, *SBP* systolic blood pressure, *SD* standard deviation, *T-chol* total cholesterol, *TG* triglycerides

Considering the significant differences in age and sex between the two groups, sex-specific and age-adjusted least mean squares were calculated for BP and HR (Table 2). No difference in research BP was found. However, both morning and evening BPs were significantly higher in the group with depressive symptoms than in the group without (male participants; morning SBP: 129.0 [95% CI, 128.0–131.0] mmHg versus 127.0 [95% CI, 126.0–128.0] mmHg; evening SBP: 126.0 [95% CI, 124.0–127.0] mmHg versus 124.0 [95% CI, 123.0–125.0] mmHg, female; morning SBP 121.0 [95%CI, 120.4–121.7] mmHg versus 119.5 [95% CI, 119.1–119.9] mmHg; evening SBP: 117.7 [95% CI, 117.0–118.3] mmHg versus 116.2 [95% CI, 115.8–116.6] mmHg). The HRs were higher in the group with depressive symptoms.

**Table 2:** Sex-specific and age-adjusted least mean squares and 95% confidence intervals for blood pressure and heart rate

		Male			Female		
		Non-depressive symptoms	Depressive symptoms	*P*-value	Non-depressive symptoms	Depressive symptoms	*P*-value
		(*n* = 1,375)	(*n* = 310)		(*n* = 3,636)	(*n* = 1,384)	
*Research*	SBP (mmHg)	124.1 (123.6–124.6)	124.4 (123.3–125.4)	0.69	118.5 (118.2–118.9)	118.4 (117.8–118.9)	0.67
	DBP (mmHg)	75.7 (75.3–76.1)	75.7 (74.8–76.5)	0.91	73.1 (72.8–73.3)	73.0 (72.6–73.4)	0.84
	HR (/min)	64.2 (63.7–64.8)	65.4 (64.3–66.5)	0.074	66.8 (66.5–67.1)	67.2 (66.6–67.6)	0.25
*Morning*	SBP (mmHg)	127.0 (126.0–128.0)	129.0 (128.0–131.0)	0.010	119.5 (119.1–119.9)	121.0 (120.4–121.7)	<0.001
	DBP (mmHg)	75.6 (75.2–76.0)	76.7 (75.8–77.7)	0.028	70.9 (70.7–71.2)	71.7 (71.2–72.1)	0.005
	HR (/min)	66.1 (65.6–66.5)	67.9 (66.9–68.9)	<0.001	67.4 (67.2–67.7)	68.4 (68.0–68.8)	<0.001
*Evening*	SBP (mmHg)	124.0 (123.0–125.0)	126.0 (124.0–127.0)	0.035	116.2 (115.8–116.6)	117.7 (117.0–118.3)	<0.001
	DBP (mmHg)	71.1 (70.7–71.6)	72.2 (71.2–73.1)	0.048	67.8 (67.5–68.1)	68.5 (68.1–68.9)	0.008
	HR (/min)	70.2 (69.7–70.7)	71.0 (70.0–72.1)	0.14	69.0 (68.8–69.2)	69.8 (69.4–70.2)	<0.001

*DBP* diastolic blood pressure, *HR* heart rate, *MHT* masked hypertension, *SBP* systolic blood pressure

The prevalence of MHT and the ORs and 95%CIs for MHT in those with depressive symptoms are shown in Table 3. Although the prevalence of MHT was higher in the group with depressive symptoms in both sexes, the difference between the two groups were large in male (male participants: 40.6% [126/310] versus 33.3% [458/1,375] and female ones: 19.3% [267/1,384] versus 17.8% [648/3,636]). The crude model revealed significant association between depressive symptoms and MHT only in male participants. After adjusting for multiple variables, the OR was 1.72 (95% CI, 1.26–2.34) in male participants and 1.30 (95% CI, 1.06–1.59) for model 3 (*P* for interaction = 0.021).

**Table 3:** Prevalence of masked hypertension and odds ratios and 95% confidence intervals for masked hypertension in the participants with depressive symptoms

	All			Male			Female		
	Non-depressive symptoms	Depressive symptoms	*P*–value	Non-depressive symptoms	Depressive symptoms	*P*-value	Non-depressive symptoms	Depressive symptoms	*P*-value
	(*n* = 5,011)	(*n* = 1,694)		(*n* = 1,375)	(*n* = 310)		(*n* = 3,636)	(*n* = 1,384)	
MHT, *n* (%)	1,106 (22.1)	393 (23.2)		458 (33.3)	126 (40.6)		648 (17.8)	267 (19.3)	
Crude, OR (95%CI)	1.00 (Ref)	1.07 (0.93–1.22)	0.34	1.00 (Ref)	1.37 (1.06–1.76)	0.014	1.00 (Ref)	1.10 (0.94–1.29)	0.23
Model 1, OR (95%CI)	1.00 (Ref)	1.41 (1.22–1.62)	<0.001	1.00 (Ref)	1.54 (1.19–1.20)	0.001	1.00 (Ref)	1.35 (1.14–1.60)	<0.001
Model 2, OR (95%CI)	1.00 (Ref)	1.42 (1.22–1.66)	<0.001	1.00 (Ref)	1.54 (1.14–2.06)	0.004	1.00 (Ref)	1.36 (1.13–1.65)	0.001
Model 3*, OR (95%CI)	1.00 (Ref)	1.41 (1.19–1.66)	<0.001	1.00 (Ref)	1.72 (1.26–2.34)	<0.001	1.00 (Ref)	1.30 (1.06–1.59)	0.012

*CI* confidence interval, *OR* odds ratio

^*^P for interaction between depressive symptoms and sex = 0.021

model 1 in all included age and sex; model 1 in male and female included age; model 2 included model 1, BMI, research SBP, research HR, treated HT, HbA1c, low–density lipoprotein cholesterol, smoking status, alcohol intake, NaCl intake, and regular exercise; and model 3 included model 2, use of sleeping pills, extent of house damage, educational status, season of the examination date, and examination year

Table 4 shows the prevalence of MHT and the ORs and 95%CIs for MHT in depressive symptoms according to age and sex. In both sex, *p* for interaction between depressive symptoms and age was not statistically significant. Moreover, it showed that age did not modify the association between MHT and depressive symptoms.

**Table 4:** Prevalence of depressive symptoms and masked hypertension, and odds ratios and 95% confidence intervals for masked hypertension in the participants with depressive symptoms according to age and sex

	Q1	Q2	Q3	Q4	
*Male*	(*n* = 441)	(*n* = 481)	(*n* = 354)	(*n* = 409)	
age (years)	≤50	≥51, ≤64	≥65, ≤69	≥70	
depressive symptoms, *n* (%)	118 (26.8)	29 (18.6)	69 (16.6)	94 (14.9)	
MHT, *n* (%)	109 (24.7)	50 (32.1)	149 (35.8)	276 (41.1)	
OR for MHT (95%CI)	1.78 (0.97–3.23)	1.50 (0.83–2.71)	1.55 (0.65–2.50)	1.28 (0.65–2.5)	*p* for interaction 0.20
*Female*	(*n* = 1,325)	(*n* = 1,298)	(*n* = 1,272)	(*n* = 1,125)	
age (years)	≤45	≥46, ≤57	≥58, ≤65	≥66	
depressive symptoms, *n* (%)	453 (34.2)	372 (28.7)	300 (23.6)	259 (23.0)	
MHT, *n* (%)	60 (4.5)	152 (11.7)	290 (22.8)	413 (36.7)	
OR for MHT (95%CI)	1.42 (0.74–2.67)	1.06 (0.67–1.65)	1.38 (0.97–1.97)	1.29 (0.91–1.81)	*p* for interaction 0.65

*CI* confidence interval, *MHT* masked hypertension, *OR* odds ratio, *Q1* the first quartile, *Q2* the second quartile, *Q3* the third quartile, *Q4* the fourth quartile

ORs and 95%CIs for MHT were calculated using model 3 including body mass index, research SBP, research HR, treated HT, HbA1c, low–density lipoprotein cholesterol, smoking status, alcohol intake, NaCl intake, regular exercise, use of sleeping pills, extent of house damage due to the Great East Japan Earthquake, educational status, season of the examination date, and year of the examination date

Table 5 shows the subgroup analysis according to research SBP, treated HT, and medical history of depression. The OR in males without treated HT was higher than in those with it (OR, 2.03 [95%CI, 1.40–2.92] versus 0.92 [0.51–1.66], *P* for interaction = 0.024). The association between depression and MHT was consistent according to other categories.

**Table 5:** Odds ratios and 95% confidence intervals for masked hypertension in the participants with depressive symptoms according to research systolic blood pressure, treated hypertension, and history of depression

		Non-depressive symptoms MHT, *n* (%)	Depressive symptoms MHT, *n* (%)	ORs (95%CIs) for MHT in depressive symptoms	*P-*value	*P* for interaction
*Male*						
*Research SBP*	SBP ≤ 125	126/677 (18.6)	48/164 (29.1)	2.09 (1.29–3.35)	0.003	0.71
	SBP > 125	332/698 (47.6)	78/145 (53.8)	1.47 (0.98–2.24)	0.064	
*Treated HT*	without	264/1016 (26.0)	86/235 (36.6)	2.03 (1.40–2.92)	<0.001	0.024
	with	194/359 (54.0)	40/75 (53.3)	0.92 (0.51–1.66)	0.78	
*History of depression*	without	447/1347 (33.2)	116/291 (39.9)	1.71 (1.24–2.35)	<0.001	0.71
	with	11/26 (42.3)	10/19 (52.6)	1.17 (0.61–1.93)	0.91	
*Female*						
*Research SBP*	SBP ≤ 119	82/1793 (4.6)	50/771 (6.5)	1.29 (0.82–2.02)	0.26	0.26
	SBP > 119	76/1738 (4.4)	46/755 (6.1)	1.30 (1.04–1.64)	0.023	
*Treated HT*	without	420/3177 (13.2)	176/1221 (14.4)	1.25 (0.98–1.58)	0.065	0.64
	with	228/459 (49.7)	91/163 (55.8)	1.37 (0.91–2.09)	0.13	
*History of depression*	without	635/3547 (17.9)	242/1254 (19.3)	1.29 (1.04–1.59)	0.019	0.53
	with	13/89 (14.6)	25/130 (19.2)	1.69 (0.54–5.59)	0.37	

*CI* confidence interval, *HT* hypertension, *MHT* masked hypertension, *OR* odds ratio, *SBP* systolic blood pressure

ORs and 95% CIs were calculated using model 3 including age, body mass index, research systolic blood pressure, research heart rate, treated hypertension (except for analysis stratified with treated HT), HbA1c, low–density lipoprotein cholesterol, smoking status, alcohol intake, NaCl intake, regular exercise, use of sleeping pills, educational status, extent of house damage due to the Great East Japan Earthquake, season of the examination date, and year of the examination date

## Discussion

The present study assessed the association between depressive symptoms and MHT in a large cohort of participants with normotension using research BP. Our results indicated that the participants with depressive symptoms had a higher prevalence of MHT, thereby supporting our hypothesis. The association was prominent in male participants.

Previous studies reported the association of WCHT with depression, anxiety, mental stress, and introverted personality traits [9, 10, 12, 29]. Nonetheless, few studies have evaluated the association between MHT and depression. Although the Finn-Home study showed that MHT was associated with depression, the analysis for depression did not include enough covariates [14]. Another study including patients with treated HT reported that the risk of depression was approximately 7-fold higher in the MHT group than in the controlled HT group [15]. The findings of our study were consistent with the results of these studies, except for in male participants with treated HT. Of note, our study analyzed in details the association between MHT and depressive symptoms using a larger sample size than did these previous studies (6705 in this study; 1459 and 328 in previous studies, respectively) and included participants with and without treated HT.

One possible mechanism underlying the association between depressive symptoms and MHT may be autonomic nervous system dysregulation. Considerable evidence exists regarding autonomic nervous system dysregulation in patients with depression [30]. Catecholamine levels, which reflect sympathetic activity, are higher in patients with depression than in healthy controls [30]. The HR is regulated by both the sympathetic and parasympathetic nervous systems. Additionally, autonomic nervous system dysfunction leads to a reduction in HR variability [31]. A frequency-domain analysis of HR variability showed sympathetic arousal and reduced parasympathetic activity in individuals with depressive symptoms [32]. In our study, the HRs in the research center and at home were higher in the participants with depressive symptoms after adjusting for age and sex, suggesting hyper sympathetic activity [30]. This might have increased the home BP and caused MHT.

With respect to the BP level categories, the sympathetic nerve activity was greater in the participants with MHT, sustained HT, and WCHT than in normotensive individuals [33]. Isolated nocturnal HT, which overlaps with MHT, is related to excess sympathetic activation due to nocturnal activity and sleep apnea [34–36]. Hyper sympathetic activity is common in patients with depression and MHT.

Diurnal BP variability and BP response to stress may partially explain our results. Increased diurnal BP variability and less nocturnal BP dipping have been reported in people with mental illnesses, including depression [32, 37, 38], and are caused by reduced baroreflex sensitivity [39]. A previous study showed that the white-coat effect, defined as BP in office minus BP at rest, was associated with an enhanced BP response to mental stress [10]. Stress triggers depression [40], and BP responses to stress may be affected by depression.

Another mechanism may be related to cortisol, an HT-inducing adrenocortical hormone [41–43]. A previous study showed that serum cortisol levels were significantly higher in participants with depression; as depressive symptoms became severe, cortisol levels increased [44]. Cortisol has diurnal variation, and may thus cause diurnal BP variation and MHT [43].

The interaction effect between sex and depressive symptoms was observed, and depressive symptoms were associated with MHT more strongly in male than in female patients. A previous study showed that depression was related to cardiovascular events only in male patients [45]. Sex differences were also reported in depression [46]. The prevalence of depression is twice as high in female patients [46], and gender differences in somatic symptoms related to depression have been reported [47]. Although the mechanism of our result is unclear, the sex differences in depression may be related.

On the other hands, association between depressive symptoms and MHT was not observed in male participants with treated HT. Treated HT itself is a risk factor for MHT: those with treated HT, particularly, male patients often have other risk factors, including obesity, smoking, and drinking [1–4, 8]. These factors may weaken the effect of depressive symptoms on MHT.

Although significant association in the participants with medical history of depression was not found due to a small sample size, sub-analysis showed a constant result regardless of medical history of depression. Previous study suggested that antidepressants could affect BP and the effect could be different between types of antidepressants [48, 49]. Our results may indicate that current depressive symptoms were important risk factor for MHT.

The present study has several limitations. First, depressive symptoms were assessed using the CES-D, which is used for screening depression but not for diagnosing depression. Second, individuals with severe depression were not included in this study because even participants with depressive symptoms could visit the Community Support Center to undergo examinations and regularly measure their home BP. However, it remains unclear whether our results are applicable to individuals with severe depression. Third, data on anti-hypertensive drugs were not available. Therefore, we could not consider the number of anti-hypertensive drugs in the analysis. Fourth, most participants were considered to have experienced the Great East Japan Earthquake [18], which might have influenced their mental status and HT management. Nevertheless, we adjusted for the extent of house damage and the examination year to reduce the effect of the Great East Japan Earthquake. In addition, information such as lifestyle, medical history, use of sleeping pills, and educational background are based on self-report questionnaires and may be misclassified. Lastly, this was a cross-sectional study and could not show a causal relationship between depressive symptoms and MHT. However, it may be reasonable that depressive symptoms may cause MHT via activation of the sympathetic nervous system, cortisol, weight gain, and diabetes [50]. We are conducting a prospective cohort study to evaluate the relationship between depressive symptoms and MHT.

## Perspective of Asia

According to World Health Organizations, the estimated prevalence of depression in the global population in 2015 is estimated to be 4.4%, and the rate in Asia is as high as that of global population [51]. However, the rate of depression is increasing. Similarly, burden of HT and its complication will increase in Asia according to aging society.

As shown in our findings, assessment of depressive symptoms in clinical settings might reveal MHT, even if their clinic BP is within normal range. This might contribute to reduce incidence of CVD or mortality in Asia.

## Conclusion

In conclusion, depressive symptoms were associated with MHT in individuals with normotension using research BP, and the association was prominent in male participants. Our study suggested that depressive symptoms may be one of the risk factors for MHT.

## References

[1] Umemura S, Arima H, Arima S, Asayama K, Dohi Y, Hirooka Y (2019). The Japanese Society of Hypertension guidelines for the management of hypertension (JSH 2019). Hypertens Res.

[2] Williams B, Mancia G, Spiering W, Agabiti Rosei E, Azizi M, Burnier M (2018). 2018 Practice guidelines for the management of arterial hypertension of the European Society of Hypertension and the European Society of Cardiology: ESH/ESC Task Force for the Management of Arterial Hypertension. J Hypertens.

[3] Unger T, Borghi C, Charchar F, Khan NA, Poulter NR, Prabhakaran D (2020). 2020 International Society of Hypertension global hypertension practice guidelines. Hypertension.

[4] Whelton PK, Carey RM, Aronow WS, Casey DE, Collins KJ, Dennison Himmelfarb C (2018). 2017 ACC/AHA/AAPA/ABC/ACPM/AGS/APhA/ASH/ASPC/NMA/PCNA guideline for the prevention, detection, evaluation, and management of high blood pressure in adults: a report of the American College of Cardiology/American Heart Association Task Force on Clinical Practice Guidelines. Hypertension.

[5] Tientcheu D, Ayers C, Das SR, McGuire DK, de Lemos JA, Khera A (2015). Target organ complications and cardiovascular events associated with masked hypertension and white-coat hypertension: analysis from the Dallas Heart Study. J Am Coll Cardiol.

[6] Brguljan-Hitij J, Thijs L, Li Y, Hansen TW, Boggia J, Liu YP (2014). Risk stratification by ambulatory blood pressure monitoring across JNC classes of conventional blood pressure. Am J Hypertens.

[7] Pickering TG, Gerin W, Schwartz JE, Spruill TM, Davidson KW (2008). Franz Volhard lecture: should doctors still measure blood pressure? The missing patients with masked hypertension. J Hypertens.

[8] Diaz KM, Veerabhadrappa P, Brown MD, Whited MC, Dubbert PM, Hickson DA (2015). Prevalence, determinants, and clinical significance of masked hypertension in a population-based sample of African Americans: the Jackson Heart Study. Am J Hypertens.

[9] Nemcsik-Bencze Z, Kőrösi B, Gyöngyösi H, Batta D, László A, Torzsa P (2022). Depression and anxiety in different hypertension phenotypes: a cross-sectional study. Ann Gen Psych.

[10] Lantelme P, Milon H, Gharib C, Gayet C, Fortrat JO (1998). White coat effect and reactivity to stress: cardiovascular and autonomic nervous system responses. Hypertension.

[11] Landsbergis PA, Dobson M, Koutsouras G, Schnall P (2013). Job strain and ambulatory blood pressure: a meta-analysis and systematic review. Am J Pub Health.

[12] Hozawa A, Ohkubo T, Obara T, Metoki H, Kikuya M, Asayama K (2006). Introversion associated with large differences between screening blood pressure and home blood pressure measurement: the Ohasama study. J Hypertens.

[13] Niu K, Hozawa A, Awata S, Guo H, Kuriyama S, Seki T (2008). Home blood pressure is associated with depressive symptoms in an elderly population aged 70 years and over: a population-based, cross-sectional analysis. Hypertens Res.

[14] Hänninen MR, Niiranen TJ, Puukka PJ, Mattila AK, Jula AM (2011). Determinants of masked hypertension in the general population: the Finn-Home study. J Hypertens.

[15] Kayano H, Koba S, Matsui T, Fukuoka H, Kaneko K, Shoji M (2015). Impact of depression on masked hypertension and variability in home blood pressure in treated hypertensive patients. Hypertens Res.

[16] Scalco AZ, Scalco MZ, Azul JB, Lotufo Neto F (2005). Hypertension and depression. Clin (Sao Paulo).

[17] Wirz-Justice A (2008). Diurnal variations of depressive symptoms. Dialogues Clin Neurosci.

[18] Hozawa A, Tanno K, Nakaya N, Nakamura T, Tsuchiya N, Hirata T (2021). Study profile of the Tohoku Medical Megabank Community-Based Cohort Study. J Epidemiol.

[19] Sakuma M, Imai Y, Nagai K, Watanabe N, Sakuma H, Minami N (1997). Reproducibility of home blood pressure measurements over a 1-year period. Am J Hypertens.

[20] Imai Y, Satoh H, Nagai K, Sakuma M, Sakuma H, Minami N (1993). Characteristics of a community-based distribution of home blood pressure in Ohasama in northern Japan. J Hypertens.

[21] Radloff LS (1977). The CES-D Scale: a self-report depression scale for research in the general population. Appl Psychol Meas.

[22] Vilagut G, Forero CG, Barbaglia G, Alonso J (2016). Screening for depression in the general population with the Center for Epidemiologic Studies Depression (CES-D): a systematic review with meta-analysis. PLoS One.

[23] Wada K, Tanaka K, Theriault G, Satoh T, Mimura M, Miyaoka H (2007). Validity of the Center for Epidemiologic Studies Depression Scale as a screening instrument of major depressive disorder among Japanese workers. Am J Ind Med.

[24] Sakurai K, Nishi A, Kondo K, Yanagida K, Kawakami N (2011). Screening performance of K6/K10 and other screening instruments for mood and anxiety disorders in Japan. Psychiatry Clin Neurosci.

[25] Usuzaki T, Ishikuro M, Metoki H, Murakami K, Noda A, Ueno F (2020). Comparison among research, home, and office blood pressure measurements for pregnant women: the TMM BirThree Cohort Study. J Clin Hypertens (Greenwich).

[26] Tanaka T, Okamura T, Miura K, Kadowaki T, Ueshima H, Nakagawa H (2002). A simple method to estimate populational 24-h urinary sodium and potassium excretion using a casual urine specimen. J Hum Hypertens.

[27] Narita K, Hoshide S, Kanegae H, Kario K (2021). Seasonal variation in masked nocturnal hypertension: the J-HOP Nocturnal Blood Pressure Study. Am J Hypertens.

[28] Øverland S, Woicik W, Sikora L, Whittaker K, Heli H, Skjelkvåle FS (2019). Seasonality and symptoms of depression: a systematic review of the literature. Epidemiol Psych Sci.

[29] Terracciano A, Scuteri A, Strait J, Sutin AR, Meirelles O, Marongiu M (2014). Are personality traits associated with white-coat and masked hypertension. J Hypertens.

[30] Carney RM, Freedland KE, Veith RC (2005). Depression, the autonomic nervous system, and coronary heart disease. Psychosom Med.

[31] Metelka R (2014). Heart rate variability–current diagnosis of the cardiac autonomic neuropathy. A review. Biomed Pap Med Fac Univ Palacky Olomouc Czech Repub.

[32] Davydov DM, Shapiro D, Cook IA, Goldstein I (2007). Baroreflex mechanisms in major depression. Prog Neuropsychopharmacol Biol Psych.

[33] Grassi G, Seravalle G, Trevano FQ, Dell’oro R, Bolla G, Cuspidi C (2007). Neurogenic abnormalities in masked hypertension. Hypertension.

[34] Fan HQ, Li Y, Thijs L, Hansen TW, Boggia J, Kikuya M (2010). Prognostic value of isolated nocturnal hypertension on ambulatory measurement in 8711 individuals from 10 populations. J Hypertens.

[35] Agarwal R, Light RP, Bills JE, Hummel LA (2009). Nocturia, nocturnal activity, and nondipping. Hypertension.

[36] Hla KM, Young T, Finn L, Peppard PE, Szklo-Coxe M, Stubbs M (2008). Longitudinal association of sleep-disordered breathing and nondipping of nocturnal blood pressure in the Wisconsin Sleep Cohort Study. Sleep.

[37] Shahimi NH, Lim R, Mat S, Goh CH, Tan MP, Lim E (2022). Association between mental illness and blood pressure variability: a systematic review. Biomed Eng Online.

[38] Otsuka K, Yamanaka G, Shinagawa M, Murakami S, Yamanaka T, Shibata K (2004). Chronomic community screening reveals about 31% depression, elevated blood pressure and infradian vascular rhythm alteration. Biomed Pharmacother.

[39] Ramirez AJ, Bertinieri G, Belli L, Cavallazzi A, Di Rienzo M (1985). Reflex control of blood pressure and heart rate by arterial baroreceptors and by cardiopulmonary receptors in the unanaesthetized cat. J Hypertens.

[40] Kendler KS, Karkowski LM, Prescott CA (1999). Causal relationship between stressful life events and the onset of major depression. Am J Psych.

[41] Whitworth JA, Brown MA, Kelly JJ, Williamson PM (1995). Mechanisms of cortisol-induced hypertension in humans. Steroids.

[42] Kelly JJ, Mangos G, Williamson PM, Whitworth JA (1998). Cortisol and hypertension. Clin Exp Pharm Physiol Suppl.

[43] Holt-Lunstad J, Steffen PR (2007). Diurnal cortisol variation is associated with nocturnal blood pressure dipping. Psychosom Med.

[44] Jia Y, Liu L, Sheng C, Cheng Z, Cui L, Li M (2019). Increased serum levels of cortisol and inflammatory cytokines in people with depression. J Nerv Ment Dis.

[45] Kabutoya T, Hoshide S, Davidson KW, Kazuomi K (2018). Sex differences and the prognosis of depressive and nondepressive patients with cardiovascular risk factors: the Japan Morning Surge–Home Blood Pressure (J-HOP) study. Hypertens Res.

[46] Alexopoulos GS (2005). Depression in the elderly. Lancet.

[47] Salk RH, Hyde JS, Abramson LY (2017). Gender Differences in Depression in Representative National Samples: Meta-Analyses of Diagnoses and Symptoms. Psychol Bull.

[48] Fu W, Ma L, Zhao X, Li Y, Zhu H, Yang W (2015). Antidepressant medication can improve hypertension in elderly patients with depression. J Clin Neurosci.

[49] Licht CMM, Penninx BWJH, de Geus EJC (2009). Response to depression and blood pressure control: All antidepressants are not the same. Hypertension.

[50] Graham N, Smith DJ (2016). Comorbidity of depression and anxiety disorders in patients with hypertension. J Hypertens.

[51] World Health Organization. Depression and Other Common Mental Disorders. https://iris.who.int/bitstream/handle/10665/254610/WHO-MSD-MER-2017.2-eng.pdf Accessed 28 September (2023)

